# Clinical Value of Serum Thrombospondin-2 Combined with CA19-9 in Early Diagnosis of Gastric Cancer

**DOI:** 10.1155/2021/2483964

**Published:** 2021-10-07

**Authors:** Lanzhi Li, Jie Dong, Lei Fu, Xinhua Xia, Feng Pan, Yuan Ning

**Affiliations:** ^1^Health Examination Department, Yantaishan Hospital, Yantai 64000, China; ^2^Department of Clinical Laboratory, Yantai Yuhuangding Hospital Affiliated To Qingdao University, Yantai 264000, China; ^3^PIVAS, Affiliated Qingdao Central Hospital, Qingdao University, Qingdao 266000, China; ^4^Ward Department, Zhangqiu District People's Hospital, Jinan 250200, China; ^5^Supply Support Center, The Central Hospital Affiliated to Shandong First Medical University, 105 Jiefang Road, Jinan 250013, Shandong, China

## Abstract

Gastric cancer (GC) is a kind of common cancer worldwide. Too late in diagnosis results in poor prognosis of patients with GC. Thrombospondin-2 (THBS2) is a type of secreted protein that has been found to be a diagnostic biomarker in a variety of cancers. Our study aimed to uncover the clinical value of THBS2 in early detection for patients with gastric cancer. THBS2 was upregulated in gastric cancer tissue compared with normal tissue via analyzing data obtained from The Cancer Genome Atlas (TCGA) database. Additionally, the enzyme-linked immunosorbent assay revealed that the level of serum THBS2 and carcinoembryonic antigen, CA19-9, was higher dramatically in patients with early gastric cancer (EGC) than that in healthy control (HC) in addition to patients with benign gastric tumor (BGT), which suggested that THBS2 indeed associated with GC. Receiver operator characteristic (ROC) curve assay was conducted to demonstrate that serum THBS2 was similar to CA19-9 to distinguish patients with early gastric cancer from healthy control and patients with benign gastric tumor and that THBS2 combined with CA19-9 improved the detective performance of THBS2 for early gastric cancer. Furthermore, we applied the gene set enrichment analysis assay to analyze signaling pathways related to THBS2. We found that THBS2 positively controlled MAPK and WNT signaling pathways, which indicated that THBS2 might exert its functions via the pathway mentioned above. Thus, our study expounded that serum THBS2 could serve as a vital early diagnostic marker for patients with gastric cancer.

## 1. Introduction

Gastric cancer (GC) as the fourth leading tumor results in third most main death rate that is related to cancers in the world [1]. The disease incidence and mortality of GC maintains high in both East Asian and Central South American countries including China, Korea, Mexico, and Chile [2, 3]. Early gastric cancer can invade either mucosa or submucosa, which is accompanied by metastasis of the lymph node or not [4]. Late detection, in addition to insensitivity to existing treatment options, causes a poor prognosis of GC [5]. For an optimum period of therapy, it is necessary to diagnose GC as early as possible. Recently, advanced technologies such as screening are employed to raise diagnosis proportion of early gastric cancer [6]. Carcinoembryonic antigen, CA19-9, and other detection methods are available to predict GC [7]. Serum markers are easier to detect than other test indexes; therefore, they are used widely to diagnose early gastric cancer [8]. The most sensitive serum index for diagnosing GC, however, is still uncovered [9]. Thus, it is urgent to explore new and highly effective biomarkers to boost the detective accuracy of early GC [10].

Thrombospondins (THBSs), a family belonging to Ca^2+^ binding glycoproteins, are secretions of immune cells and mesenchymal cells in addition to endotheliocytes [11]. They can bind to plenty of downstream proteins involved in various biological procedures, including cell migration, blood vessel production, apoptosis, and cytoskeletal regulation [12, 13]. In multiple malignancies, THBS2 is tight correlated with progression and prognosis of cancers [14]. Tokunaga et al. discovered that the expression of THBS2 in colon cancer decreased hepatic metastases risk as well as angiogenesis of tumors compared to patients lacking THBS2 [15]. Additionally, THBS2 had been found to exert a vital impact on lung adenocarcinoma, prostate cancer, myeloid tumor, and breast cancer [16, 17]. Nevertheless, the specific effect of THBS2, in addition to its clinical value for gastric cancer, is not disclosed now. Therefore, we are trying to expose its clinical significance in the detection of early gastric cancer.

## 2. Materials and Methods

### 2.1. Patients and Samples

This is a prospective study. Blood was obtained from 41 healthy individuals, 33 benign gastric tumor patients, and 46 early gastric cancer patients at Yantaishan Hospital, Yantai, China. The benign or early stage of cancer was confirmed by the principle of the American Joint Committee on Cancer (AJCC) TNM (tumor–node–metastasis) classification. Blood was centrifuged; then, serum was collected and stored at −80°C freezer. Basic information such as age, gender, and amounts of samples are given in Table 1. All participants voluntarily participated in our studies, and all protocols related to human volunteers were abiding by the guidance of the Helsinki Declaration. All patients provided written informed consent, and the study was approved by the Ethics Committee of the Yantaishan Hospital, Yantai, China.

### 2.2. Gene Set Enrichment Analysis (GSEA)

To verify the molecular signaling pathway related to the high expression of THBS2, GSEA was conducted by using GSEA 3.0 software. Specific gene sets were downloaded from the official website (http://software.broadinstitute.org/gsea/index.jsp) and analyzed for pathway enrichment.

### 2.3. Enzyme-Linked Immunosorbent Assay (ELISA)

The protein levels of THBS2 and CA19-9 in serum were examined by the ELISA kit (ThermoFisher, USA). Serum from early gastric cancer (EGC), benign gastric tumor (BGT), or healthy control (HC) was incubated with a microtiter plate which had been treated with capture antibodies for 1 hour at room temperature. Then, the plate was washed and incubated with a specific antibody for 0.5 hour at room temperature, followed by washing the plate. Then, the secondary antibody was added into plate, and reaction was terminated by a stop solution. A microplate reader (BioRad, CA) was employed to record the absorbance at 450 nm. The detailed protocol was in accordance with the reagent specification.

### 2.4. Statistical Analysis

All data were analyzed by using SPSS 19.0 software (IBM, USA) and GraphPad 8.0 software (CABIT, China) for calculating statistical significance and was presented in a manner of mean ± standard deviation (SD). The correlation between THBS2 and CA19-9 was measured by Pearson's correlation analysis. Receiver operator characteristic (ROC) curves were drawn to estimate the diagnostic performance. All relevant data are given in Table 2. *P* < 0.05 is statistically significant.

## 3. Results

### 3.1. The Level of THBS2 Is Increased in Gastric Cancer Tissues

**Figure 1:**
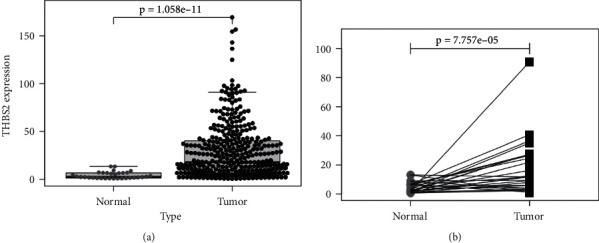
The level of THBS2 increased in gastric cancer tissues. (a) Data from TCGA website are deferential analyzed. (b) Data from TCGA website are paired deferential analyzed.

The load of THBS2 in tumor tissues and normal tissues of patients diagnosed with gastric cancers was analyzed by deferential analysis as well as paired differential analysis on the basis of data from The Cancer Genome Atlas (TCGA) database. The mean level of THBS2 in gastric cancer tissues was obviously higher than that in control tissues (*P* < 0.001, Figures 1(a) and 1(b)). The data showed that serum THBS2 was potential to be secreted by gastric cancer tissues.

### 3.2. Increased Level of THBS2 in Serum of Patients with Early Gastric Cancer

**Figure 2:**
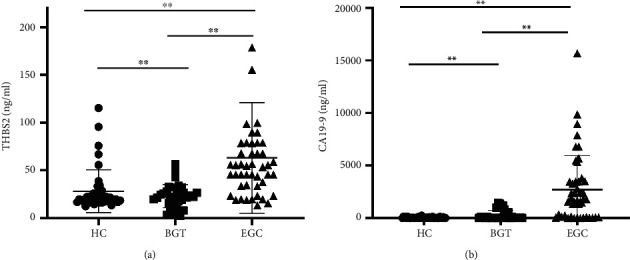
Increased level of THBS2 in serum of patients with early gastric cancer. (a) The level of serum THBS2 in various samples measured by the ELISA assay (*P* < 0.05). (b) The level of serum CA19-9 in various samples measured by the ELISA assay (*P* < 0.05).

To confirm results mentioned above, an ELISA was conducted to measure the load of serum THBS2 in 46 patients with EGC or 33 patients with BGT in addition to 41 HC. The results illustrated that the protein level of serum THBS2 in people with EGC was upregulated dramatically compared with that in HC and BGT patients. Additionally, the load of serum THBS2 in BGT patients was higher than that in HC (*P* < 0.05, Figure 2(a)). CA19-9, a common biomarker for GC, was also estimated by the ELISA assay. As shown in Figure 2(b), the level of serum CA19-9 was the highest in EGC patients while the lowest in HC, which is consistent with the load of serum THBS2 (*P* < 0.05, Figure 2(b)).

### 3.3. The Correlation between Serum THBS2 and CA19-9

**Figure 3:**
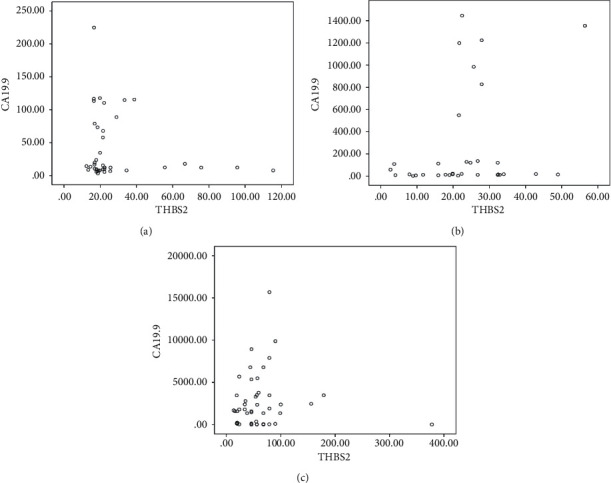
The correlation between serum THBS2 and CA19-9. (a) The correlation between THBS2 and CA19-9 in HC. (b) The correlation between THBS2 and CA19-9 in patients with BGT. (c) The correlation between THBS2 and CA19-9 in patients with EGC.

To reveal how serum THBS2 influences the detection of early gastric cancer, we made scatter plots based on the level of serum THBS2 in addition to CA19-9 and analyzed their relationship. Interestingly, there was no obvious correlation between THBS2 and CA19-9 in health controls sample (*P*=0.157) as well as benign patients sample (*P*=0.292), while there existed a significant correlation in patients with early gastric cancer (*P*=0.04) (Figures 3(a)–3(c)).

### 3.4. The Detected Performance of THBS2 and CA19-9 for Early Gastric Cancer

**Figure 4:**
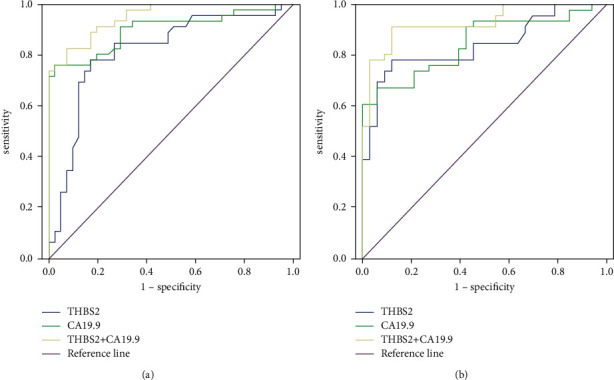
The detected performance of THBS2 and CA19-9 for early gastric cancer. (a) Diagnostic performance of THBST in addition to CA19-9 to differentiate early gastric cancer patients from a healthy control. (b) Diagnostic performance of THBST in addition to CA19-9 to differentiate early gastric cancer patients from benign gastric tumor patients.

ROC curve analysis was employed to explore the performance of early diagnosis about serum THBS2 and CA19-9 in patients with GC (Figure 4(a)). Additionally, we listed all data obtained from the above analysis, including area under the curve (AUC) and asymptotic 95% confidence interval in Table 2. Serum THBS2 possessed well capacity to forecast early gastric cancer with the value of AUC: 0.816 (95% CI: 0.722–0.911). Serum CA19-9 distinguished patients with EGC from healthy control with the value of AUC of 0.901 (95% CI: 0.833–0.968). Furthermore, serum THBS2 combined with CA19-9 as a marker could promote the performance of THBS2 or CA19-9 as an individual index (AUC: 0.951, 95% CI: 0.912–0.989). Subsequently, we conducted the ROC curve to uncover whether THBS2 and CA19-9 differentiated patients with EGC from patients with BGT. As shown in Figure 4(b), the data showed that THBS2 or CA19-9 was able to predict early gastric cancer as an individual biomarker and combined THBS2 with CA19-9 could improve the diagnostic performance dramatically. All detailed data are given in Table 2.

### 3.5. THBS2 Plays a Vital Role in GC via Potentially Regulating MAPK and WNT Signaling Pathway

**Figure 5:**
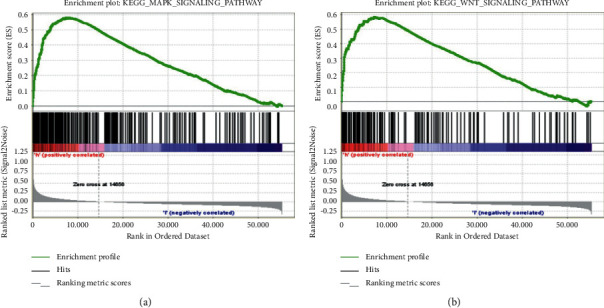
THBS2 playing a vital role in GC via potentially regulating MAPK and WNT signaling pathways. (a) Genes related to MAPK signaling pathway positively enriched by THBS2. (b) Genes related to WNT signaling pathway positively enriched by THBS2.

To further explore the functional mechanism of serum THBS2 on GC, we conducted the GSEA assay with data from the TCGA database and analyzed the THBS2-related pathway. The GSEA enrichment plot showed that high THBS2 expression was positively enriched in mitogen-activated protein kinase (MAPK) as well as Wnt-*β*/catenin signaling pathway and exhibited a tightly positive relationship with multiple downstream genes involved in the pathway as mentioned earlier (Figures 5(a) and 5(b)).

## 4. Discussion

Increasing novel technologies, including liquid biopsy, have been employed to diagnose cancers; however, various proteins lack sensitivity in addition to specificity for applying into clinical practice [17]. More blood-based markers such as serum proteins possess the ability to offer information that is associated with the progression of cancers timely [18]. Therefore, serum biomarkers related to the early stage of cancers are required to diagnose and follow up patients with malignancy. Recently, emerging studies have revealed various markers that possess potential to detect gastric cancer in the early stage. For example, serum amyloid A cluster (SAA) and high mobility group box 1 (HMGB1) can be regarded as significant biomarkers for early diagnosis of GC [19]. Thymidine kinase 1 (TK1), CA19-9, and CA72-4 combined with other carcinoembryonic antigen exhibited better detection ability of GC and colorectal cancer (CRC) [20].

THBS2, one of the proteins belonging to thrombospondin family, has been reported to serve as a vital regulator of tumorigenesis [21]. In multiple cancers, decreasing the expression of THBS2 can trigger production of oncogenes or depress production of tumor suppresser genes. Upregulating THBS2 in cancer tissues is correlated to inhibit progression of tumors sometimes [15, 22, 23]. Simpson RE et al. found that THBS2 was a biomarker of pancreatic ductal adenocarcinoma and related to a high rate of dysplasia in sufferers with premalignant symptoms [24]. Liu et al. reported that it accelerates lung cancer progression through producing matrix metalloproteinase-13 [25]. In addition, serum THBS2 has been regarded as a valuable clinical marker for various cancers. For example, THBS2 has the ability to forecast prognosis for patients with colorectal cancer [26]. Downregulating THBS2 exerts utterly opposite functions on the clinical outcome of sufferers with gastric cancer [27, 28]. Nevertheless, no enough research studies devoted into uncovering the clinical value of serum THBS2 for diagnosing early gastric cancer.

**Figure 6:**
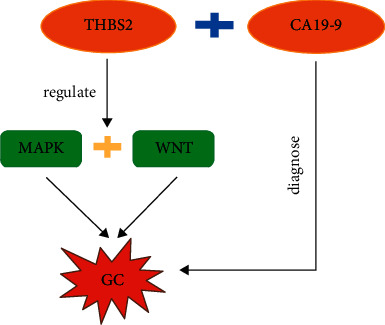
Schematic diagram of THBS2 regulating GC and THBS2 combined CA19-9 existing better performance in diagnosing GC.

In our present study, we first analyzed data from the TCGA database and found that THBS2 was upregulated dramatically in gastric cancer tumor, which indicated that THBS2 might exert a crucial role in GC. To confirm the hypothesis, we collected tissue samples from 41 cases of HC and 33 cases of BGT in addition to 46 cases of EGC to measure the serum load of THBS2 by using ELISA assay. The results demonstrated that the level of serum THBST was upregulated in patients with EGC compared to patients with BGT as well as HC significantly, implying that the increase of THBS2 was an important marker of GC. According to previous studies, the performance will get better to combine THBS2 and CA19-9 in detecting pancreatic ductal adenocarcinoma and distal cholangiocarcinoma [29]. Bamlet et al. discovered that THBS2 had good detection performance in tumors combined with CA19-9 [30]. Chen et al. revealed that THBS2 as one of biomarkers could be replenished by CA19-9 for diagnosing colorectal cancer [31]. Given these reporters, we assumed THBS2 combined CA19-9 may exert better performance in diagnosing GC. Subsequently, we further conducted the ELISA assay and found the load of CA19-9 in serum of patients diagnosed with EGC was higher than that in EGT as well as in HC, which illustrated that cancer tissues were likely to produce more THBS2 in serum in accordance with former research studies [32, 33]. In addition, there exhibited an obvious correlation between THBS2 and CA19-9 in patients with ECG (*P*=0.04) but not in HC (*P*=0.157) and patients with EGT (*P*=0.292) according to correlation analysis, indicating that both THBS2 and CA19-9 play an essential role in diagnosis of early GC. Next, ROC curve analysis was employed to reveal that THBS2 possessed a comparable ability to differentiate patients with EGC from HC and patients with EGT to CA19-9. Additionally, the detective capacity was notably improved when we combined THBS2 and CA19-9 as the diagnostic marker. Moreover, GSEA was employed to uncover that THBS2 positively related with the MAPK and Wnt-*β*/catenin signaling pathway, implying the potential regulatory mechanism of serum THBS2 on gastric cancer first, eventhough it was also necessary to conduct more experiments to explore the detailed mechanisms (Figure 6).

There are still some limitations to our study. For example, our investigation did not conduct sufficient experiments to explore the detailed functions of THBS2 combined CA19-9 for GC in vivo. Moreover, we have not identified the direct target gene for THBS2. Additionally, more attention need to be paid to identify that whether THBS2 combined with CA19-9 exert equally dramatic effects on early detection of other cancers.

## 5. Conclusion

In conclusion, our study uncovered that THBS2 was upregulated in GC tissues and positively regulated MAPK in addition to the WNT signaling pathway. Moreover, both THBS2 and CA19-9 could serve as significant and excellent biomarkers for detecting GC in the early stage. Additionally, THBS2 combined CA19-9 exhibited better performance in the diagnosis of early GC, which may improve diagnostic efficiency for patients with GC.

## Figures and Tables

**Table 1:** Basic information.

Diagnosis	Healthy control	Benign gastric tumor	Early gastric cancer
Sex			
Male	22	15	25
Female	19	18	21

Age			
Mean	55	53	58
Range	21–67	20–65	24–71
Amount	41	33	46

**Table 2:** Data of ROC curve.

	AUC	95% CI
HC vs. EGC		
THBS2	0.816	0.722–0.911
CA19.9	0.901	0.833–0.968
THBS2 + CA19.9	0.951	0.912–0.989

BGT vs. EGC		
THBS2	0.840	0.752–0.927
CA19.9	0.847	0.762–0.931
THBS2 + CA19.9	0.928	0.872–0.984

^*∗*^ *P* < 0.05 in comparison with CA19.9.

## Data Availability

The data used to support the findings of this study are included within the article.
